# Screening for Postpartum Depression and Associated Factors Among Women in China: A Cross-Sectional Study

**DOI:** 10.3389/fpsyg.2016.01668

**Published:** 2016-11-01

**Authors:** Xinli Chi, Peichao Zhang, Haiyan Wu, Jian Wang

**Affiliations:** ^1^College of Psychology and Sociology, Shenzhen University, ShenzhenChina; ^2^Research Center of Modern Psychology, Wuhan University, WuhanChina; ^3^State Grid Anhui Maintenance Company, HefeiChina; ^4^College of politics and law, Anhui Jianzhu University, HefeiChina

**Keywords:** postpartum depression, postnatal depression, prevalence, correlates, adult attachment

## Abstract

**Objectives:** This study examined what percentage of Chinese mothers during a three-year postpartum period were screened for postpartum depression and explored the correlation between postpartum depression and various socio-demographic, psychological, and cultural factors.

**Study design:** Cross-sectional survey.

**Methods:** A total of 506 mothers 23 years of age and older who were within three years postpartum completed the online survey. The survey collected information such as family economic status, a history of depression, preparation for pregnancy, relationships with husbands, and family members, adult attachment types (Adult Attachment Scale, AAS), and depression (The Center for Epidemiologic Studies Depression Scale, CESD).

**Results:** Approximately 30% of mothers 1–3 years postpartum reported symptoms above the CESD cut-off score (≥16 scores) associated with the risk for depression (28.0% in the first year, 30.8% in the second year, and 31.8% in the third year). Factors significantly associated with depression in participants in the correlation analysis were education level; family income; preparation for pregnancy; a history of depression; amount of time spent with their husbands; relationships with husbands, parents, and parents-in-law; and a close, dependent, and/or anxious attachment style. Multiple regression analyses revealed that a history of depression; less preparation for pregnancy; poorer relationships with husbands, parents, and parents-in-law; and a more anxious attachment style were strongly related to a higher risk of postpartum depression.

**Conclusion:** The overall percentage of mothers after delivery who were vulnerable to depression in China remains high. Various factors were significant predictors of postpartum depression. The research findings have several valuable implications for intervention practices. For example, attachment styles and depression history in the assessments of perinatal depression could improve screenings and the design of interventions. Additionally, improving the family relationships and family environments of women post-delivery may be promising approach for postpartum depression prevention or intervention.

## Introduction

Depression is one of the most prominent mental disorders in women post-delivery; it is correlated with adverse consequences for mothers, children, and families (e.g., poor maternal mental health and adverse cognitive development in children) (Glasheen et al., 2010; Martini et al., 2015). Maternal postpartum depression has become a significant public concern. Although there are numerous studies examining the prevalence and correlates of postpartum depression in mothers from many countries, to date, such investigations have been inadequate in China. As such, the present research was designed to investigate what percentage of Chinese mothers during a three-year postpartum period were screened for postpartum depression and potential correlates of postpartum depression. This will help to more precisely target prevention and intervention for mothers and children at risk.

Prior studies based in Western countries have examined the prevalence of postpartum depression. Prevalence rates varied widely across countries and regions. For example, a review study found the mean prevalence of postpartum depression was 15.4% in the United States, 38.1% in Italy, 17.1% in Germany, and 22.5% in Ireland (Halbreich and Karkun, 2006). In a study involving 6,421 women between five and 14 months postpartum in Canada, the overall prevalence of postpartum depression was 8.0% in the 12 weeks prior to the study (Simone Vigod, 2012). Another study of 100 primiparous women in Australia found that at 12 months postpartum, 30% of all mothers reported clinically significant levels of depressive symptomatology (McMahon et al., 2005).

In the Asian context, several important prevalence studies have been conducted. In Japan, of 70 Japanese mothers who were assessed by psychiatrists, Ueda et al. (2006) found that 27% mothers at 12 months postpartum were diagnosed as having experienced a new onset of depression. In Taiwan, Wang et al. (2003) conducted a study with 315 mothers six weeks post-delivery and found that about 31 and 12.9% mothers had experienced mild-to-moderate and moderate-to-severe depression, respectively. In a recent study including 2,072 postpartum women in Malaysia, Yusuff et al. (2015) reported that 14.3% of mothers were categorized as having depression within the first 6 months postpartum. A study done in Tianjin, China indicated that the prevalence of postpartum depression was 10.2% among 463 mothers 35–60 days post-delivery (Zhang et al., 2001).

Regarding factors associated with maternal postpartum depression, existing studies have examined predictors from socio-demographic, psychological, and cultural perspectives. For example, Schmied et al. (2013) reported that mothers’ past depression or existing mental disorders were associated with an increased risk of postpartum depression. Prior researchers have also found that lower maternal education level and poor family economic status were related to a higher prevalence of postpartum depression (Schmied et al., 2013). Empirical literature has consistently demonstrated that poor relationships with husbands or family members (i.e., relationships with mothers and/or mothers-in-law) are linked to a greater likelihood of women experiencing postpartum depressive symptoms post-delivery (Leung et al., 2005; Saligheh et al., 2014; Martini et al., 2015). Furthermore, studies have shown that a lack of psychological preparedness for pregnancy and insecure attachment styles to partners (i.e., avoidance and anxiety) are associated with a higher risk of postpartum depression (Sabuncuoǧlu and Berkem, 2006; Martin et al., 2014). Moreover, there is a preference for giving birth to a boy in some Asian societies, and the association between infant’s sex and postpartum depression has attracted scholars’ interest. Several studies based in Asian contexts (e.g., India, Hong Kong, Vietnam) have shown that the delivery of a baby girl led to a greater risk of postpartum depression among women compared to the delivery of a baby boy (Rodrigues et al., 2003; Xie et al., 2007).

Although prior studies provide valuable information, most were conducted in highly developed countries and regions (e.g., USA and Taiwan) and there is little research examining what percentage of women during a three-year postpartum period were screened for postpartum depression is in developing countries, such as China. In addition, the majority of studies to date were carried out on women in their first year postpartum, so what percentage of women were vulnerable to depression remains unknown after the first year of the postpartum period. Some researchers have noted that postpartum depression can continue or occur in the second and third years post-delivery (Garthus-Niegel et al., 2015). Third, while various correlates of postpartum depression are well established, research on factors that contribute to postpartum depression is still scarce in China. Fourth, most prior studies investigated selected correlates from only one or two main categories (e.g., socio-demographic); factors from multiple categories (e.g., socio-demographic, psychological and cultural) have rarely been examined in a single study. These gaps in previous studies of postpartum depression signal a need for research that can shed light on what percentage of Chinese mothers during a three-year postpartum period were screened for depression and multiple predictors of postpartum depression.

The present study was thus designed to address two primary research questions: (a) How prevalent is depression in Chinese mothers during their first three years postpartum? (b) what correlates (i.e., mothers’ education, family economic status, preparation for pregnancy, past depression experience, relationship with family members, and babies’ age, sex, number and health, and adult attachment) are associated with depression in mothers during this three-year postpartum period?

## Materials and Methods

### Participants

Data for the current study were collected online in China from July to October 2015. A total of 550 mothers 23 years of age and over with children from 0 to 3 years old (0–36 months) voluntarily participated in the survey. In total, 506 mothers completed the survey. Further socio-demographic information for the participants is presented in **Table 1**.

**Table 1:** Socio-demographic characteristics of the study.

Variables	*N*	*%*
**Education**		
>9 years	465	91.9
≤9 years	41	8.1
**Residence**		
Village	198	39.1
Town	110	21.7
City	198	39.1
**Family income**		
Lower than average level	17	3.4
Average level	355	70.2
Higher than middle level	134	26.5
**Preparation for parenthood**		
None	124	24.5
Not enough	254	50.2
Enough	128	25.3
**Depression experience(≥3 months)**		
No	253	50.0
Maybe	136	26.9
Yes	117	23.1
**Babies’ sex**^∗^		
Male	251	49.6
Female	255	50.4
**Babies’ age**		
≤1 year old	193	38.1
1~2 years old	175	34.6
2~3 years old	138	27.3
**Numbers of babies**		
One child	400	79.1
Two and more children	106	20.9
**Babies’ health**		
Not good	15	3.0
Good	491	97
**Enough time with you husband spent**		
Yes	308	60.9
No	198	39.1
**Marital relationship satisfaction**		
Very satisfied	117	23.1
Somewhat satisfied	241	47.6
Neither satisfied nor dissatisfied	105	20.8
Dissatisfied	26	5.1
Very dissatisfied	17	3.4
**Relationship with parents**		
Very satisfied	204	40.3
Somewhat satisfied	184	36.4
Neither satisfied nor dissatisfied	92	18.2
Dissatisfied	22	4.3
Very dissatisfied	4	0.8
**Relationship with parents in law**		
Very satisfied	75	14.8
Somewhat satisfied	188	37.2
Neither satisfied nor dissatisfied	172	34.0
Dissatisfied	48	9.5
Very dissatisfied	23	4.5


*^∗^If more than two children, “sex” for the youngest.*

*Mothers (*N* = 506).*

### Procedure

The survey was conducted via an online mental health questionnaire system at Anhui University of Architecture. During the survey period, the link to the questionnaire was repeatedly distributed via multiple chat platforms popular with women after delivery, including QQ chat groups, Wechat groups^1^, and the bulletin board system (BBS) about mothers, birth, and childrearing. We invited mothers with children from 0 to 3 years old to respond to the online questionnaire. Before responding to the questionnaire, participants had to register and obtain a name and password. Subsequently, they used their name and password to log in to the system to complete the questionnaire. The registration system designation ensured that participants felt that their data would be kept confidential and that they would only participate in the survey once. The purposes of the survey and the confidentiality of the data collected were thoroughly highlighted in the instructions. Clear directions on how to complete the questionnaire were also provided at the beginning of the survey. After the mothers completed the online survey, their data were saved automatically in the database of the Information Technology System (ITS). Meanwhile, upon completion of the questionnaire, an online payment of 15 RMB was automatically sent to them as remuneration.

### Ethics Statement

This study was approved by the Human Research Ethics Committee at Anhui University of Architecture. Informed written consent was obtained from participants before the assessment. Participants were also told that they were free to withdraw from the study at any time during the data collection.

### Measures

#### Depression

The Center for Epidemiologic Studies Depression Scale (CES-D) was used to measure participants’ depression symptoms and to detect people at risk of experiencing a depressive disorder. The 20-item CES-D asked participants to respond on a four-point Likert scale (0 = “rarely or none of the time,” 3 = “most or all of the time”) indicating whether they had experienced the listed depression symptoms in the prior week. The sum of the response scores ranged from 0 to 60; higher scores indicated more severe depressive symptoms. Participants who scored less than 16 were classified as non-depressed, and those who scored over 16 (CES-D ≥ 16) were classified as depressed (Feng et al., 2010; Jewkes et al., 2010). Prior studies on peripartum depression have shown that the CES-D has good psychometric properties (Tandon et al., 2012; Park et al., 2015). The Cronbach’s alpha and Spearman-Brown coefficients of CES-D scores for the present sample were 0.72 and 0.81, respectively, which indicate good internal consistency and reliability.

#### Correlates

Participants were asked to report on their education level (0 ≤*9 years*, 1 ≥*9 years*), residence (0 = *village*, 1 = *town*, 2 = *city*), and family income (0 = *lower than average level*, 1 = *average level*, 2 = *higher than average level*). They reported whether they had sufficient preparation for pregnancy (0 = *none*, 1 = *not enough*, 2 = *enough*), and whether they had a history of depression (≥3 months) (0 = *no*, 1 = *maybe*, 2 = *yes*). Participants also reported babies’ sex (0 = male, 1 = female), babies’ age (0 = *0*–*1 year old*, 1 = *1*–*2 years old*, 2 = *2*–*3 years old*), their total number of children (0 = *one child*, 1 = *two or more children*), and babies’ health (0 = *not good*, 1 = *good*). In addition, participants reported whether their husbands had sufficient time to stay with them during their pregnancy and birth (0 = *yes*, 1 = *no*), whether they were satisfied with their relationships with their husbands, parents, and parents-in-law (0 = *very satisfied*, 1 = *somewhat satisfied*, 2 = *neither satisfied and nor dissatisfied*, 3 = *dissatisfied*, 4 = *very dissatisfied*).

#### Adult Attachment Styles

Participants’ adult attachment styles, considered one of the correlates, were investigated using Collins and Read’s (1990) adult attachment scale (AAS), with 18 items assessing three attachment dimensions (closeness, dependence, and anxiety). The closeness dimension measures the extent to which a person is comfortable with closeness and intimacy. The dependence subscale measures the extent to which a person feels s/he can depend on others to be available when needed. The anxiety subscale measures the extent to which a person is worried about being abandoned or unloved. These items were answered using five-point scales ranging from 1 (“not at all characteristic of me”) to 7 (“very characteristic of me”). Existing research showed that the AAS has sound psychometric properties (Collins and Feeney, 2004; Mallinckrodt and Wei, 2005; Kane et al., 2007). For the current sample, the Cronbach’s alpha and Spearman-Brown coefficients of the AAS for the present sample were 0.72 and 0.77, respectively, which indicate good internal consistency and reliability.

### Data Analysis Plan

First, frequencies and percentages were computed for all independent variables and participants’ reported depression levels (see **Table 1**). Second, bivariate correlation analyses were carried out to examine how the multiple factors correlated with the participants’ reported levels of depression. Third, multiple regression analyses were performed to explore the predictive effects of participants’ factors on their depression levels. The variables that were significantly associated with depression in bivariate correlation analyses were treated as the independent variables, and participants’ reported depression levels were considered the dependent variables. All ordinal-level and continuous independent variables were standardized with *z*-transformation. Categorical variables were dummy-coded as 0 (male) and 1 (female). All analyses were performed using SPSS for Windows, version 22.0. In the interpretation of the results, the statistical significance was set at *p* < 0.05 (two-tailed).

## Results

### Screening for Postpartum Depression

The overall percentage of mothers who were found to be at risk of experiencing depressive symptoms during the 3-year postpartum period was 30% (152/506). Specifically, for the first year postpartum, the percentage of mothers who were detected at risk of experiencing depressive symptoms was 28.0% (54/193); for the second year postpartum it was 30.8% (54/175), and for the third year postpartum it was 31.8% (44/138).

### Correlates with Postpartum Depression

In bivariate correlation analysis, 11 variables were observed to be significantly correlated with higher levels of depression (*p* < 0.01): lower education level; lower family income; less preparation for pregnancy; a history of depression; less time spent with their husbands; worse relationships with husbands, parents, and parents-in-law; and more close, dependent, and anxious attachment styles. The other variables, including residence, babies’ sex, babies’ health and age, and total number of children were non-significant factors for depression (*p* > 0.05) (**Table 2**).

**Table 2:** Bivariate correlation analysis of various independent variables and depression.

	Depression (CESD)
Education level	-0.127^∗∗^
Residence	-0.030
Family income	-0.153^∗∗^
Preparation for parenthood	-0.191^∗∗^
Depression experience (>=3 months)	0.437^∗∗^
Babies’ sex	-0.29
Babies’ health	-0.043
Babies’ age	-0.007
Number of babies	-0.043
Enough time with you husband spent	0.235^∗∗^
Marital relationship satisfaction	0.423^∗∗^
Relationship with parents	0.362^∗∗^
Relationship with parents in law	0.362^∗∗^
Close attachment	0.163^∗∗^
Dependent attachment	0.326^∗∗^
Anxious attachment	0.581^∗∗^


*^∗∗^*p* < 0.01*

### Predictors of Postpartum Depression

As can be seen in **Table 3**, the above 11 significant variables were selected to be included in a multiple regression model. Preparation for pregnancy; a history of depression; relationship with husbands, parents, and parents-in-law; and an anxious attachment style emerged as significant predictors of participants’ postpartum depression. Specifically, mothers who had less preparation for pregnancy were more depressed than those who had sufficient preparation (β = -0.07, *p* = 0.040). Mothers who had a history of depression were also more depressed than mothers who had no prior history (β = 0.23, *p* = 0.000). Mothers who had poorer relationships with their husbands, parents, and parents-in-law were more depressed than those who had better relationships with their husbands (β = 0.13, *p* = 0.003), parents (β = 0.09, *p* = 0.023), and parents-in-law (β = 0.08, *p* = 0.051).

**Table 3:** Multiple lineal regression of various independent variables on depression.

Independent variables	*B*	*SE*	β	*t*	△*R*^2^
**Total explanatory power**					0.449^∗∗∗^
Education level	-1.86	1.32	-0.49	-1.40	
Family income	-0.26	0.74	-0.01	-0.35	
Preparation for parenthood	-1.03	0.52	-0.07	-2.00^∗^	
Depression experience (≥3 months)	2.96	0.47	0.23	6.26^∗∗∗^	
Depression experience of own mothers (≥3 months)	0.08	1.29	0.01	0.07	
Enough time with you husband spent	-0.89	0.84	-0.04	-1.05	
Marital relationship	1.46	0.49	0.14	3.01^∗∗^	
Relationship with parents	0.83	0.42	0.07	1.96^∗^	
Relationship with parents in law	0.83	0.41	0.08	2.03^∗^	
Close attachment	-0.08	0.93	-0.01	-0.09	
Dependent attachment	0.23	0.91	0.01	0.25	
Anxious attachment	4.82	0.58	0.39	8.32^∗∗∗^	


*^∗^*p* < 0.05; ^∗∗^*p* < 0.01; ^∗∗∗^*p* < 0.001.*

## Discussion

The purpose of the current study was to investigate what percentage of Chinese mothers during their first three years postpartum were screened for postpartum depression. The study also examined various socio-demographic, psychological, and cultural correlates of depression in these mothers. The results showed that an average of 30.0% (ranging from 28.0 to 31.8%) of the participating mothers experienced a high level of depression in the first three years postpartum, which indicates that the percentage of women post-delivery in China at risk for depression remains high. Our findings are similar to those of studies conducted in other regions, such as Australia (30.0%) (McMahon et al., 2005), Japan (27.0%) (Ueda et al., 2006), and Taiwan (31.0%) (Wang et al., 2003); however, we found a lower rate of depression than those prior researchers found in Tianjin (10.2%) (Zhang et al., 2001) and Henan province (20.9%) (Guo et al., 2003), China.

This may be due to the different sampling methods utilized in these studies. First, participants in the present study were drawn from various parts of China, not only one city or province, which may explain the higher prevalence rate in the present study. Second, we employed a web survey to collect data. This approach may reduce social desirability effects (Heerwegh, 2009), as in our online survey, participants were completely anonymous. This may have made it easier for them to find the appropriate time and place to complete the questionnaire alone. This could have made them feel safer and therefore more willing to express their true feelings and ideas (Wright, 2005). In addition, the different depression scales used in the various studies, including the CES-D, Beck Depression Inventory (BDI), Edinburgh Postnatal Depression Scale (EPDS), and Zung Self-Rating Depression Scale (SDS), may in part explain the variation between these studies because, as noted by Shafer (2006), there are fewer common specific depression symptom factors across these depression scales than expected. However, The Center for Epidemiologic Studies Depression Scale (CESD) has high discriminative and internal consistency based on a general population sample (Van Dam and Earleywine, 2011) compared to other depression scales, including multiple dimensions such as depressed mood, feelings of guilt and worthlessness, feelings of helplessness and hopelessness, psychomotor retardation, loss of appetite, and sleep disturbance (Radloff, 1977). Furthermore, an online questionnaire system was utilized for collecting data in this study. These features may make the findings relatively comparable.

As expected, most of the factors investigated in this study, including education attachment, family income, preparation for pregnancy, relationships with family members, and adult attachment style, were correlated with depression in mothers post-delivery. However, only some correlates showed significant predictive effects in our multiple regression model. A history of depression; less preparation for parenthood; lower satisfaction with their relationships with their husbands, parents, and parents-in-law; and more anxious attachment style were linked to more depressive symptoms.

First, we found that an anxious attachment style had the strongest association with a high level of postpartum depression in women. These findings are largely consistent with prior research (e.g., Robertson et al., 2004; Martin et al., 2014). There are two possible explanations for these findings. One is that an intimate relationship with one’s baby in the postpartum period may evoke internal anxiety and conflicts, which are components of the insecure attachment style (i.e., anxious attachment) of the mother, and can thus result in depressive emotions (Sabuncuoǧlu and Berkem, 2006). The other may be that the transition to parenthood can trigger mothers’ feelings of anxiety, or lead to experiences such as receiving inadequate love and emotional support from husbands and family members, which may in turn increase the likelihood of depressive symptoms (Simpson et al., 2003). Our findings highlight the importance of maternal attachment styles in the postpartum period. A history of depression had the second strongest association with increased depressive symptoms, which was consistent with evidence from existing research that a prior history of depression puts women at risk for postpartum depression (Johnstone et al., 2001). Our results indicate that women with a history of depression should be aware that this may potentially place them in a vulnerable group.

The quality of women’s family relationships, specifically lower levels of satisfaction with their relationships with their husbands, parents, and parents-in-law, was significantly correlated with higher levels of postpartum depression. It is understandable that an unsupportive relationship with her husband can trigger stress and feelings of anxiety and helplessness in a woman post-delivery, which may incur an increased risk of postpartum depression. Moreover, in Chinese culture, when their son or daughter has a child of their own, it is common for parents to join their son/daughter’s family to help take care of their daughter/daughter-in-law and grandchild, which can create conflicts between family members (Chan et al., 2002; Murray et al., 2015). This potential increase in family stress or conflict can increase depressive symptoms in postpartum women (Murray et al., 2015). Existing research has indicated that intergenerational conflicts, particularly in-law conflicts, are a risk factor for maternal postpartum depression in Asian families (Chan et al., 2002; Lee et al., 2004).

We also found that less preparation for parenthood was associated with increased depressive symptoms. Prior research has demonstrated that unplanned pregnancy and a lack of preparation for parenthood were significantly linked to higher levels of depression in postpartum mothers (Cox et al., 2008). The transition to parenthood is a stressful and difficult life event. Inadequate preparation for pregnancy, childbirth, and nursing may lead mothers to feel anxious and helpless and have less (or no) ability to cope with all the changes and challenges babies bring, which accordingly may increase the likelihood of depressive disorders in mothers (Spiteri et al., 2014). Our findings indicate that interventions or programs to help with the preparation for parenthood should be developed and implemented for Chinese women who plan to give birth.

Contrary to our expectations, our results indicated that the health, number, age, and sex of babies were not significant predictors of women’s levels of postpartum depression. Notably, the finding that there was no association between the sex of their children and mothers’ depression in this study was inconsistent with some prior studies (Lee et al., 2000; Patel et al., 2002). However, it was in line with a recent study showing that the preference for a boy was not linked to depressive symptoms in mothers (Murray et al., 2015). There are three possible reasons for this contradictory finding. First, it is possible that along with the influence of Western culture and the development of Asian societies, including China, people, particularly members of the younger generation, are gradually changing their attitude toward female children (Purewal, 2010). Another explanation may be the one-child policy that has been implemented throughout the past three decades in China. Each couple has only been allowed to have one child, no matter whether that child was a boy or a girl, which may have led to a decline in the preference for male children (Chi et al., 2013). The third reason could be our sample composition: only mothers with a computer or smartphone were able to participate in our online survey. Mothers in rural areas may have fewer electronic devices or less internet access than urban mothers, which may have resulted in more urban mothers and fewer rural mothers in our sample. This sampling bias may make this association insignificant, as prior research has found that the preference for having boys is stronger in rural areas (Hatlebakk, 2012). Future studies could be conducted specifically in rural areas to examine the possible associations between a child’s sex and a mother’s postpartum depression.

Another finding that was inconsistent with our expectations. Namely, the amount of time participants’ husbands spent with them during their pregnancy and delivery was not significantly related to their postpartum depression levels. There are two potential explanations for this insignificant finding. First, the quality of women’s family relationships, including their relationships with their husbands, parents, and parents-in-law, may be indeed stronger predictors than the amount of time participants’ husbands spent with them during their pregnancy and delivery. While a variety of factors at familial levels were found to affect women’s postnatal depression, positive family relationships have always been regarded as the first requisite for the prevention or/and intervention postnatal depression (Fisher et al., 2012). Second, it is likely that the quality of the marital relationship and the amount of time participants’ husbands spent with them interactively correlate with participants’ postnatal depression. For example, a good marital relationship often promotes the willingness of husbands to spend time, even more time, with their wives, which in turn promotes a healthy marital relationship. A virtuous cycle may develop between a good marital relationship and sufficient company, which may thus promote mothers’ mental health level during postnatal period. Future research may focus on examining the interactive effects between family relationships, particularly the marital relationship, and the amount of time participants’ husbands spent with them during their pregnancy and delivery to identify more familial factors associated with postnatal depression.

There were several limitations in this study. First, its cross-sectional design prevented us from identifying cause-and-effect associations; we could not determine whether poor relationships with family members increased depressive symptoms in mothers or whether maternal depression led to poor family relationships. Future studies may utilize longitudinal methods to track a single group of mothers to examine the causal relationships between depression and the risk factors identified in the present study. Second, because of the nature of our self-reporting methodology, the findings in this study must be interpreted with caution. However, participants in this study were recruited online, and it is known that a high degree of privacy can reduce the possibility of dishonesty. Third, although various socio-demographic, psychological, and cultural correlates were examined in this study, prior research has shown that biological factors such as oxytocin levels, personality traits, peer support, and education experience preparing parents for the transition into parenthood are also significantly correlated with maternal postpartum depression. More relational, personal, and biological predictors may be examined in future research.

Notwithstanding these limitations, our study has several valuable implications for intervention practices in China. First, the high percentage of Chinese women who experienced a high level of depression in their postpartum period indicates that there is urgent need for developing and implementing related interventions or psychoeducational programs among them. Second, the results of the present study suggest that women with a history of depression or insecure attachment styles (i.e., anxious attachment) may be a particularly important focus of Chinese intervention strategies that aim to prevent or/and reduce maternal postpartum depression. Third, our findings suggest that mothers’ relationships with their family members, particularly the marital relationship, and their family environments are potential risk factors that should be considered in assessments of perinatal depression and the development of interventions. In addition, related intervention programs focused on improving family relationships may not only be developed targeting women having babies, but also their family members, such as husbands, parents, or parents-in law. Finally, the study suggests that in addition to face-to-face intervention programs, online intervention websites providing relevant information, education/courses, or training might also be a good choice for women and their family members to promote their well-being and foster a more positive family atmosphere.

## Author Contributions

XC designed the research, performed the statistical analysis. PZ and JW drafted manuscript. PZ and XC polished language of the manuscript. XC and HW revised the manuscript.

## Conflict of Interest Statement

The authors declare that the research was conducted in the absence of any commercial or financial relationships that could be construed as a potential conflict of interest.

## Footnotes

1 QQ and Wechat are the two most popular instant messaging service tools in China. People can chat one-on-one or within groups with up to 500 persons, using a computer or a smartphone. The Internet software is similar to MSN or Facebook.
